# Bis{(*E*)-3-[(diethyl­methyl­ammonio)meth­yl]-*N*-[3-(*N*,*N*-dimethyl­sulfamo­yl)-1-methyl­pyridin-4-yl­idene]-4-methoxy­anilinium} tetra­iodide penta­hydrate

**DOI:** 10.1107/S1600536809000324

**Published:** 2009-01-10

**Authors:** Tiago Rodrigues, Rui Moreira, Bruno Dacunha-Marinho, Francisca Lopes

**Affiliations:** a iMed.UL, Faculty of Pharmacy, University of Lisbon, Av. Prof. Gama Pinto, 1649-003 Lisbon, Portugal; b Ed. CACTUS, Campus Sur, Unidade de Raios X, Universidad de Santiago Compostela 15782, Spain

## Abstract

The title compound, 2C_21_H_34_N_4_O_3_S^2+^·4I^−^·5H_2_O, was prepared exclusively as the *E* isomer by methyl­ation of the corresponding *N*-phenyl­pyridin-4-amine. There are two symmetry-independent mol­ecules in the asymmetric unit with no significant differences in bond lengths and angles. The aromatic rings are not coplanar with the pyridin-4-imine groups, as indicated by the C—N—C—C torsion angles of 47.7 (7) and 132.6 (5)°.

## Related literature

For background information see: Bjorkman & Bhattarai (2005 ▶); Yeates *et al.* (2008 ▶). For related literature structures, see: Lopes *et al.* (2004 ▶); Wang *et al.* (2008 ▶); Djedouani *et al.* (2008 ▶).
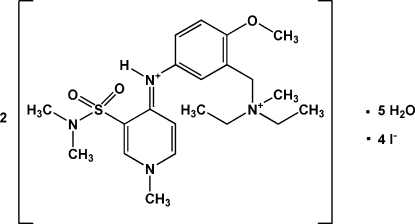


## Experimental

### 

#### Crystal data

2C_21_H_34_N_4_O_3_S^2+^·4I^−^·5H_2_O
*M* *_r_* = 1442.86
Triclinic, 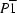

*a* = 12.7930 (5) Å
*b* = 13.5539 (6) Å
*c* = 16.8386 (7) Å
α = 96.670 (2)°
β = 97.667 (2)°
γ = 98.224 (1)°
*V* = 2836.5 (2) Å^3^
*Z* = 2
Mo *K*α radiation
μ = 2.33 mm^−1^
*T* = 100 (2) K
0.35 × 0.2 × 0.08 mm

#### Data collection

Bruker APEXII CCD diffractometer
Absorption correction: multi-scan (*SADABS*; Bruker, 2005 ▶) *T*_min_ = 0.575, *T*_max_ = 0.830
49577 measured reflections
11468 independent reflections
8753 reflections with *I* > 2σ(*I*)
*R*_int_ = 0.050

#### Refinement

*R*[*F* ^2^ > 2σ(*F* ^2^)] = 0.043
*wR*(*F* ^2^) = 0.116
*S* = 1.00
11468 reflections
654 parameters
17 restraints
H atoms treated by a mixture of independent and constrained refinement
Δρ_max_ = 2.07 e Å^−3^
Δρ_min_ = −1.42 e Å^−3^

### 

Data collection: *APEX2* (Bruker, 2005 ▶); cell refinement: *SAINT* (Bruker, 2005 ▶); data reduction: *SAINT*; program(s) used to solve structure: *SIR97* (Altomare *et al.*, 1999 ▶); program(s) used to refine structure: *SHELXL97* (Sheldrick, 2008 ▶); molecular graphics: *ORTEP-3 for Windows* (Farrugia, 1997 ▶); software used to prepare material for publication: *WinGX* (Farrugia, 1999 ▶).

## Supplementary Material

Crystal structure: contains datablocks global, I. DOI: 10.1107/S1600536809000324/bg2227sup1.cif

Structure factors: contains datablocks I. DOI: 10.1107/S1600536809000324/bg2227Isup2.hkl

Additional supplementary materials:  crystallographic information; 3D view; checkCIF report

## Figures and Tables

**Table 1:** Hydrogen-bond geometry (Å, °)

*D*—H⋯*A*	*D*—H	H⋯*A*	*D*⋯*A*	*D*—H⋯*A*
O1*W*—H1*WA*⋯I4	0.91 (3)	2.60 (4)	3.489 (4)	170 (3)
O1*W*—H1*WB*⋯I2	0.90 (2)	2.663 (17)	3.561 (4)	176 (4)
O2*W*—H2*WA*⋯O5w^i^	0.90 (4)	1.98 (5)	2.799 (7)	147 (5)
O2*W*—H2*WB*⋯I3	0.90 (4)	2.69 (4)	3.580 (5)	175 (4)
O3*W*—H3*WA*⋯O1*W*^i^	0.90 (4)	1.91 (4)	2.781 (6)	161 (18)
O3*W*—H3*WB*⋯I1	0.90 (4)	2.64 (4)	3.544 (4)	178 (5)
O4*W*—H4*WA*⋯I4	0.90 (3)	2.75 (3)	3.613 (5)	160 (4)
O4*W*—H4*WB*⋯O3*W*^ii^	0.89 (5)	1.92 (4)	2.795 (7)	162 (2)
O5*W*—H5*WA*⋯I2	0.90 (4)	2.75 (5)	3.614 (5)	165 (4)
O5*W*—H5*WB*⋯O4w^iii^	0.90 (4)	1.94 (4)	2.809 (7)	161 (3)
N14—H14⋯O9	0.90 (3)	1.93 (4)	2.733 (5)	149 (4)
N54—H54⋯O49	0.90 (5)	2.08 (4)	2.767 (5)	133 (4)

Symmetry codes: (i) 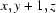; (ii) 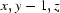; (iii) 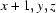.

**Fig. 1.**
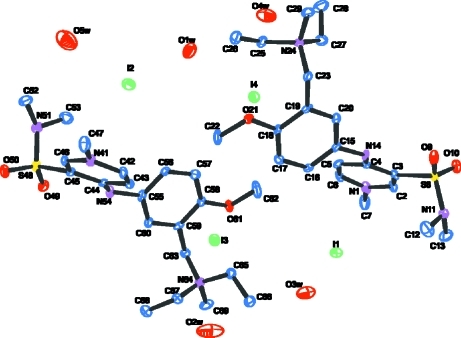
An ORTEPIII (Farrugia, 1997) view of the molecular structure of the title compound, showing the labelling of all non-H atoms. Displacement ellipsoids for non-H atoms are shown at the 50% probability level. H atoms have been omitted for clarity.

## supplementary crystallographic information

### Comment

Malaria is accounted as one of the major diseases worldwide, and for which few efficient drugs are known today [Bjorkman and Bhattarai, 2005]. 4(1*H*)-Pyridones are currently being developed as important and potential antimalarial agents, capable of inhibiting the *bc*_1_ complex, at the oxidation site (Q_o_ site) level in the *Plasmodium falciparum *mitochondrion [Yeates *et al.*, 2008]. As part of our project towards the synthesis of 4(1*H*)-pyridone bioisosteric scaffolds, the (1*H*-pyridin-4-ylidene)amine scaffold was studied.

The title compound was prepared by reaction of the corresponding *N*-phenylpyridin-4-amine with methyl iodide. Interestingly, only the *E *isomer of the compound was obtained, as it was previously observed for amodiaquine analogues [Lopes *et al.*, 2004]. There are two symmetry-independent molecules in the asymmetric unit with no significant differences in bond lengths and angles. The observed imine bond distances C4—N14 and C44—N54 are longer than the expected by *ca* 0.035 Å [Wang *et al.*, 2008 and Djedouani *et al.*, 2008], a consequence of the imine group being protonated. The aromatic rings are not coplanar relatively to the pyridin-4-imine moieties, as indicated by the C4—N14—C15—C16 and C44—N54—C55—C56 dihedral angles of 47.7 (7)° and 132.6 (5)°, respectively. The molecules are hydrogen-bonded through the imine nitrogen atoms at N14 and N54, acting as donors towards the sulfonyl oxygen atoms O9 and O19 of each sulfonamide moiety, respectively. The (1*H*-pyridin-4-ylidene)amine scaffold is nearly planar and the C5—C4—N14—C15 dihedral angle is 7.9 (7)° for one of the molecules, whereas the C43—C44—N54—C55 dihedral angle on the other molecule is -14.1 (7)°.

### Experimental

The title compound was prepared at room temperature by reacting 2-[(diethylamino)methyl]-4-(pyridin-4-ylamino)phenol with methyl iodide in the presence of NaH in DMF. Crystals were grown from water.

### Refinement

The hydroxy H atoms for the water solvent molecules were initially located in a difference Fourier map, but their distances were constrained with *DFIX* at 0.9 Å from the O atom and with DANG at 2.5 Å from the other H water atom. The hydrogen atoms linked to the charged N14 and N54 atoms were located in a difference Fourier map, but the distances N—H were constrained at 0.9 Å, in order to get the refinement stabilization. The rest of the H atoms were positioned geometrically and included as riding atoms with C—H = 0.95 or 0.98 Å and *U*_iso_(H)= 1.2 or 1.5 times *U*_eq_(C).

### Crystal data

**Table:**

2C_21_H_34_N_4_O_3_S^2+^·4I^−^·5H_2_O	*Z* = 2
*M**_r_* = 1442.86	*F*(000) = 1436.0
Triclinic, *P*1	*D*_x_ = 1.687 Mg m^−^^3^
Hall symbol: -P 1	Mo *K*α radiation, λ = 0.7107 Å
*a* = 12.7930 (5) Å	Cell parameters from 9916 reflections
*b* = 13.5539 (6) Å	θ = 2.4–25.8°
*c* = 16.8386 (7) Å	µ = 2.33 mm^−^^1^
α = 96.670 (2)°	*T* = 100 K
β = 97.667 (2)°	Prism, colourless
γ = 98.224 (1)°	0.35 × 0.2 × 0.08 mm
*V* = 2836.5 (2) Å^3^	

### Data collection

**Table:**

Bruker APEXII CCD diffractometer	8753 reflections with *I* > 2σ(*I*)
Radiation source: fine-focus sealed tube	*R*_int_ = 0.050
ω and φ scans	θ_max_ = 26.4°, θ_min_ = 1.6°
Absorption correction: multi-scan (*SADABS*; Bruker, 2005)	*h* = −15→15
*T*_min_ = 0.575, *T*_max_ = 0.830	*k* = −16→16
49577 measured reflections	*l* = 0→21
11468 independent reflections	

### Refinement

**Table:**

Refinement on *F*^2^	Primary atom site location: structure-invariant direct methods
Least-squares matrix: full	Secondary atom site location: difference Fourier map
*R*[*F*^2^ > 2σ(*F*^2^)] = 0.043	Hydrogen site location: inferred from neighbouring sites
*wR*(*F*^2^) = 0.116	H atoms treated by a mixture of independent and constrained refinement
*S* = 1.00	*w* = 1/[σ^2^(*F*_o_^2^) + (0.0655*P*)^2^] where *P* = (*F*_o_^2^ + 2*F*_c_^2^)/3
11468 reflections	(Δ/σ)_max_ = 0.002
654 parameters	Δρ_max_ = 2.07 e Å^−^^3^
17 restraints	Δρ_min_ = −1.42 e Å^−^^3^

### Special details

**Table:**

Geometry. All s.u.'s (except the s.u. in the dihedral angle between two l.s. planes) are estimated using the full covariance matrix. The cell s.u.'s are taken into account individually in the estimation of s.u.'s in distances, angles and torsion angles; correlations between s.u.'s in cell parameters are only used when they are defined by crystal symmetry. An approximate (isotropic) treatment of cell s.u.'s is used for estimating s.u.'s involving l.s. planes.
Refinement. Refinement of *F*^2^ against ALL reflections. The weighted *R*-factor *wR* and goodness of fit *S* are based on *F*^2^, conventional *R*-factors *R* are based on *F*, with *F* set to zero for negative *F*^2^. The threshold expression of *F*^2^ > 2σ(*F*^2^) is used only for calculating *R*-factors(gt) *etc*. and is not relevant to the choice of reflections for refinement. *R*-factors based on *F*^2^ are statistically about twice as large as those based on *F*, and *R*- factors based on ALL data will be even larger.

### Fractional atomic coordinates and isotropic or equivalent isotropic displacement parameters (Å^2^)

**Table:**

	*x*	*y*	*z*	*U*_iso_*/*U*_eq_	
O1W	0.4474 (3)	0.1184 (3)	0.6544 (2)	0.0347 (10)	
H1WA	0.414 (3)	0.172 (2)	0.665 (3)	0.042*	
H1WB	0.5190 (9)	0.135 (3)	0.665 (3)	0.042*	
O2W	0.8769 (5)	0.9278 (3)	0.8610 (3)	0.0587 (14)	
H2WA	0.913 (5)	0.940 (4)	0.820 (3)	0.070*	
H2WB	0.836 (5)	0.867 (2)	0.852 (3)	0.070*	
O3W	0.2931 (4)	0.9651 (3)	0.6823 (2)	0.0428 (11)	
H3WA	0.352 (3)	1.010 (3)	0.681 (3)	0.051*	
H3WB	0.299 (4)	0.931 (4)	0.725 (2)	0.051*	
O4W	0.1157 (4)	0.0625 (4)	0.6565 (3)	0.0485 (12)	
H4WA	0.145 (4)	0.1267 (15)	0.655 (4)	0.058*	
H4WB	0.166 (3)	0.026 (3)	0.674 (4)	0.058*	
O5W	0.9315 (4)	0.0196 (4)	0.7284 (3)	0.0555 (13)	
H5WA	0.892 (4)	0.065 (4)	0.713 (4)	0.067*	
H5WB	0.989 (3)	0.018 (4)	0.703 (3)	0.067*	
I1	0.32351 (3)	0.83284 (3)	0.85134 (2)	0.02456 (10)	
I2	0.73146 (3)	0.17740 (3)	0.68619 (2)	0.02986 (11)	
I3	0.71949 (3)	0.68226 (3)	0.811724 (17)	0.01851 (10)	
I4	0.28842 (3)	0.30343 (3)	0.687434 (18)	0.02097 (10)	
N1	0.0320 (3)	0.6774 (3)	0.7703 (2)	0.0179 (9)	
C2	0.0149 (4)	0.6876 (4)	0.8477 (2)	0.0171 (11)	
H2	−0.0368	0.7251	0.8623	0.021*	
C3	0.0710 (4)	0.6446 (4)	0.9058 (2)	0.0147 (10)	
C4	0.1531 (4)	0.5890 (3)	0.8865 (3)	0.0136 (10)	
C5	0.1607 (4)	0.5714 (4)	0.8024 (3)	0.0155 (10)	
H5	0.2057	0.5282	0.7846	0.019*	
C6	0.1033 (4)	0.6167 (4)	0.7484 (2)	0.0185 (11)	
H6	0.1126	0.6064	0.6943	0.022*	
C7	−0.0207 (4)	0.7323 (4)	0.7111 (3)	0.0278 (13)	
H7A	−0.0628	0.7754	0.7374	0.042*	
H7B	−0.0662	0.6853	0.6693	0.042*	
H7C	0.0323	0.7722	0.6878	0.042*	
S8	0.03896 (9)	0.67067 (9)	1.00503 (6)	0.0135 (3)	
O9	0.0913 (3)	0.6067 (3)	1.05430 (18)	0.0209 (8)	
O10	−0.0746 (3)	0.6639 (3)	0.99700 (18)	0.0204 (8)	
N11	0.0902 (3)	0.7864 (3)	1.0408 (2)	0.0175 (9)	
C12	0.2061 (4)	0.8092 (5)	1.0648 (3)	0.0321 (14)	
H12A	0.2401	0.8128	1.0175	0.048*	
H12B	0.2299	0.7573	1.0934	0.048*	
H12C	0.2245	0.8726	1.0993	0.048*	
C13	0.0461 (5)	0.8645 (4)	0.9992 (3)	0.0293 (13)	
H13A	0.0769	0.8710	0.9508	0.044*	
H13B	0.0625	0.9276	1.0343	0.044*	
H13C	−0.0301	0.8460	0.9855	0.044*	
N14	0.2167 (3)	0.5534 (3)	0.9424 (2)	0.0146 (9)	
H14	0.194 (4)	0.561 (3)	0.9901 (14)	0.017*	
C15	0.3067 (4)	0.5053 (3)	0.9282 (2)	0.0134 (10)	
C16	0.3822 (4)	0.5466 (3)	0.8849 (2)	0.0116 (10)	
H16	0.3753	0.6065	0.8643	0.014*	
C17	0.4682 (4)	0.4985 (4)	0.8721 (2)	0.0141 (10)	
H17	0.5191	0.5262	0.8429	0.017*	
C18	0.4789 (4)	0.4094 (4)	0.9026 (2)	0.0134 (10)	
C19	0.4046 (4)	0.3688 (3)	0.9500 (2)	0.0131 (10)	
C20	0.3190 (4)	0.4191 (4)	0.9622 (2)	0.0135 (10)	
H20	0.2696	0.3941	0.9936	0.016*	
O21	0.5609 (3)	0.3568 (2)	0.89193 (18)	0.0156 (7)	
C22	0.6274 (4)	0.3884 (4)	0.8348 (3)	0.0284 (13)	
H22A	0.5836	0.3918	0.7845	0.043*	
H22B	0.6751	0.3410	0.8261	0.043*	
H22C	0.6681	0.4536	0.8553	0.043*	
C23	0.4197 (4)	0.2776 (3)	0.9912 (2)	0.0140 (10)	
H23A	0.3960	0.2871	1.0436	0.017*	
H23B	0.4956	0.2745	1.0010	0.017*	
N24	0.3617 (3)	0.1764 (3)	0.9456 (2)	0.0142 (9)	
C25	0.4097 (4)	0.1478 (4)	0.8698 (3)	0.0172 (11)	
H25A	0.3627	0.0909	0.8369	0.021*	
H25B	0.4130	0.2037	0.8386	0.021*	
C26	0.5201 (4)	0.1207 (4)	0.8870 (3)	0.0238 (12)	
H26A	0.5641	0.1716	0.9267	0.036*	
H26B	0.5511	0.1162	0.8380	0.036*	
H26C	0.5154	0.0570	0.9071	0.036*	
C27	0.2446 (4)	0.1837 (4)	0.9202 (3)	0.0199 (11)	
H27A	0.2177	0.2154	0.9663	0.024*	
H27B	0.2399	0.2271	0.8784	0.024*	
C28	0.1728 (4)	0.0841 (4)	0.8887 (3)	0.0295 (13)	
H28A	0.2054	0.0462	0.8499	0.044*	
H28B	0.1049	0.0963	0.8635	0.044*	
H28C	0.1626	0.0468	0.9329	0.044*	
C29	0.3720 (4)	0.1004 (4)	1.0034 (3)	0.0193 (11)	
H29A	0.3495	0.0338	0.9745	0.029*	
H29B	0.3280	0.1116	1.0445	0.029*	
H29C	0.4452	0.1073	1.0283	0.029*	
N41	0.9817 (3)	0.3632 (3)	0.7359 (2)	0.0143 (9)	
C42	0.9073 (4)	0.4212 (4)	0.7574 (2)	0.0150 (11)	
H42	0.8988	0.4330	0.8115	0.018*	
C43	0.8464 (4)	0.4612 (4)	0.7019 (3)	0.0140 (10)	
H43	0.7975	0.5008	0.7185	0.017*	
C44	0.8558 (4)	0.4437 (3)	0.6181 (2)	0.0118 (10)	
C45	0.9372 (4)	0.3870 (3)	0.5991 (2)	0.0113 (10)	
C46	0.9962 (4)	0.3490 (3)	0.6584 (3)	0.0135 (10)	
H46	1.0484	0.3119	0.6445	0.016*	
C47	1.0393 (4)	0.3134 (4)	0.7974 (3)	0.0226 (12)	
H47A	0.9904	0.2617	0.8136	0.034*	
H47B	1.0703	0.3621	0.8436	0.034*	
H47C	1.0948	0.2840	0.7751	0.034*	
S48	0.95911 (10)	0.34819 (9)	0.49898 (6)	0.0144 (3)	
O49	0.9141 (3)	0.4153 (2)	0.44787 (17)	0.0172 (7)	
O50	1.0700 (3)	0.3398 (3)	0.50195 (18)	0.0179 (8)	
N51	0.8905 (3)	0.2370 (3)	0.4701 (2)	0.0191 (9)	
C52	0.9303 (5)	0.1519 (4)	0.5057 (3)	0.0262 (13)	
H52A	0.9004	0.1429	0.5542	0.039*	
H52B	1.0068	0.1660	0.5182	0.039*	
H52C	0.9093	0.0915	0.4676	0.039*	
C53	0.7730 (4)	0.2294 (4)	0.4602 (3)	0.0265 (13)	
H53A	0.7516	0.2823	0.4316	0.040*	
H53B	0.7505	0.2356	0.5125	0.040*	
H53C	0.7405	0.1653	0.4301	0.040*	
N54	0.7923 (3)	0.4792 (3)	0.5618 (2)	0.0145 (9)	
H54	0.813 (4)	0.486 (3)	0.5136 (14)	0.017*	
C55	0.6969 (4)	0.5173 (4)	0.5752 (2)	0.0142 (10)	
C56	0.6208 (4)	0.4609 (4)	0.6102 (2)	0.0129 (10)	
H56	0.6335	0.3998	0.6263	0.015*	
C57	0.5259 (4)	0.4951 (4)	0.6215 (2)	0.0156 (10)	
H57	0.4751	0.4571	0.6451	0.019*	
C58	0.5075 (4)	0.5860 (4)	0.5973 (2)	0.0123 (10)	
C59	0.5836 (4)	0.6442 (3)	0.5615 (2)	0.0120 (10)	
C60	0.6781 (4)	0.6071 (4)	0.5503 (2)	0.0148 (10)	
H60	0.7287	0.6438	0.5257	0.018*	
O61	0.4154 (3)	0.6249 (3)	0.60549 (18)	0.0172 (8)	
C62	0.3569 (5)	0.5914 (5)	0.6669 (3)	0.0381 (16)	
H62A	0.3151	0.5264	0.6473	0.057*	
H62B	0.3105	0.6385	0.6803	0.057*	
H62C	0.4060	0.5869	0.7143	0.057*	
C63	0.5599 (4)	0.7401 (3)	0.5315 (2)	0.0154 (10)	
H63A	0.4832	0.7341	0.5159	0.019*	
H63B	0.5925	0.7473	0.4833	0.019*	
N64	0.5981 (3)	0.8360 (3)	0.5915 (2)	0.0162 (9)	
C65	0.5493 (4)	0.8250 (4)	0.6690 (3)	0.0182 (11)	
H65A	0.4721	0.8108	0.6546	0.022*	
H65B	0.5718	0.7672	0.6912	0.022*	
C66	0.5774 (4)	0.9148 (4)	0.7346 (3)	0.0221 (12)	
H66A	0.6522	0.9236	0.7556	0.033*	
H66B	0.5363	0.9037	0.7774	0.033*	
H66C	0.5615	0.9740	0.7124	0.033*	
C67	0.7185 (4)	0.8567 (4)	0.6140 (3)	0.0192 (11)	
H67A	0.7416	0.7993	0.6366	0.023*	
H67B	0.7377	0.9144	0.6559	0.023*	
C68	0.7793 (4)	0.8771 (4)	0.5438 (3)	0.0298 (14)	
H68A	0.7636	0.8192	0.5030	0.045*	
H68B	0.8547	0.8907	0.5632	0.045*	
H68C	0.7578	0.9342	0.5212	0.045*	
C69	0.5592 (4)	0.9205 (4)	0.5531 (3)	0.0244 (12)	
H69A	0.5949	0.9835	0.5833	0.037*	
H69B	0.4835	0.9158	0.5528	0.037*	
H69C	0.5742	0.9167	0.4986	0.037*	

### Atomic displacement parameters (Å^2^)

**Table:**

	*U*^11^	*U*^22^	*U*^33^	*U*^12^	*U*^13^	*U*^23^
O1W	0.039 (3)	0.033 (3)	0.037 (2)	0.018 (2)	0.011 (2)	0.0050 (19)
O2W	0.083 (4)	0.032 (3)	0.046 (3)	−0.016 (3)	−0.019 (3)	0.008 (2)
O3W	0.059 (3)	0.027 (3)	0.037 (2)	0.000 (2)	−0.003 (2)	0.0035 (19)
O4W	0.044 (3)	0.034 (3)	0.061 (3)	−0.010 (2)	0.001 (2)	0.008 (2)
O5W	0.034 (3)	0.063 (4)	0.072 (3)	0.003 (2)	0.006 (2)	0.028 (3)
I1	0.0291 (2)	0.0148 (2)	0.03152 (18)	0.00230 (15)	0.01180 (15)	0.00385 (14)
I2	0.0374 (2)	0.0207 (2)	0.03183 (19)	−0.00250 (17)	0.01728 (17)	0.00016 (15)
I3	0.02080 (19)	0.0211 (2)	0.01620 (15)	0.00576 (15)	0.00688 (13)	0.00553 (13)
I4	0.02065 (19)	0.0215 (2)	0.02325 (16)	0.00578 (15)	0.00691 (14)	0.00628 (14)
N1	0.021 (2)	0.026 (3)	0.0111 (17)	0.009 (2)	0.0055 (16)	0.0093 (17)
C2	0.016 (3)	0.024 (3)	0.013 (2)	0.005 (2)	0.0072 (19)	0.005 (2)
C3	0.020 (3)	0.016 (3)	0.010 (2)	0.002 (2)	0.0079 (19)	0.0060 (19)
C4	0.015 (3)	0.009 (3)	0.017 (2)	−0.001 (2)	0.0066 (19)	0.0013 (19)
C5	0.013 (3)	0.010 (3)	0.020 (2)	−0.001 (2)	0.001 (2)	−0.0069 (19)
C6	0.020 (3)	0.028 (3)	0.008 (2)	0.001 (2)	0.0085 (19)	0.003 (2)
C7	0.033 (3)	0.043 (4)	0.014 (2)	0.019 (3)	0.008 (2)	0.011 (2)
S8	0.0166 (6)	0.0169 (7)	0.0104 (5)	0.0073 (5)	0.0068 (5)	0.0052 (5)
O9	0.027 (2)	0.030 (2)	0.0143 (15)	0.0194 (17)	0.0100 (14)	0.0111 (14)
O10	0.0186 (19)	0.029 (2)	0.0169 (15)	0.0085 (16)	0.0073 (14)	0.0054 (15)
N11	0.020 (2)	0.016 (2)	0.0162 (18)	0.0019 (19)	0.0064 (17)	−0.0004 (17)
C12	0.027 (3)	0.045 (4)	0.019 (2)	−0.007 (3)	0.002 (2)	0.000 (2)
C13	0.045 (4)	0.017 (3)	0.032 (3)	0.014 (3)	0.012 (3)	0.007 (2)
N14	0.020 (2)	0.017 (2)	0.0110 (17)	0.0109 (19)	0.0099 (16)	0.0027 (16)
C15	0.017 (3)	0.010 (3)	0.013 (2)	0.004 (2)	0.0037 (19)	−0.0021 (18)
C16	0.014 (2)	0.009 (3)	0.0121 (19)	0.003 (2)	0.0003 (18)	0.0009 (18)
C17	0.013 (2)	0.015 (3)	0.013 (2)	−0.001 (2)	0.0028 (18)	0.0013 (19)
C18	0.017 (3)	0.013 (3)	0.012 (2)	0.005 (2)	0.0078 (19)	−0.0003 (18)
C19	0.018 (3)	0.009 (3)	0.012 (2)	0.006 (2)	−0.0009 (19)	−0.0010 (18)
C20	0.018 (3)	0.016 (3)	0.0069 (19)	0.003 (2)	0.0046 (18)	−0.0002 (18)
O21	0.0182 (18)	0.0119 (19)	0.0200 (15)	0.0068 (15)	0.0084 (14)	0.0044 (14)
C22	0.031 (3)	0.033 (4)	0.033 (3)	0.020 (3)	0.025 (3)	0.013 (2)
C23	0.019 (3)	0.013 (3)	0.013 (2)	0.006 (2)	0.0072 (19)	0.0026 (18)
N24	0.022 (2)	0.009 (2)	0.0134 (17)	0.0030 (18)	0.0061 (16)	0.0032 (16)
C25	0.021 (3)	0.015 (3)	0.015 (2)	0.006 (2)	0.006 (2)	−0.0019 (19)
C26	0.026 (3)	0.023 (3)	0.026 (2)	0.010 (2)	0.010 (2)	0.004 (2)
C27	0.017 (3)	0.016 (3)	0.027 (2)	0.004 (2)	0.003 (2)	0.005 (2)
C28	0.027 (3)	0.018 (3)	0.040 (3)	0.001 (3)	0.002 (3)	−0.006 (2)
C29	0.027 (3)	0.012 (3)	0.020 (2)	0.005 (2)	0.005 (2)	0.004 (2)
N41	0.015 (2)	0.018 (2)	0.0118 (17)	0.0056 (18)	0.0040 (16)	0.0040 (16)
C42	0.018 (3)	0.019 (3)	0.011 (2)	0.006 (2)	0.0092 (19)	0.0050 (19)
C43	0.011 (2)	0.013 (3)	0.019 (2)	0.003 (2)	0.0057 (19)	0.0019 (19)
C44	0.016 (3)	0.008 (2)	0.013 (2)	0.003 (2)	0.0039 (19)	0.0030 (18)
C45	0.017 (3)	0.010 (3)	0.0083 (19)	0.003 (2)	0.0049 (18)	0.0022 (17)
C46	0.012 (2)	0.013 (3)	0.019 (2)	0.005 (2)	0.0088 (19)	0.0037 (19)
C47	0.024 (3)	0.034 (3)	0.016 (2)	0.014 (3)	0.008 (2)	0.012 (2)
S48	0.0197 (7)	0.0166 (7)	0.0100 (5)	0.0068 (5)	0.0079 (5)	0.0029 (5)
O49	0.024 (2)	0.017 (2)	0.0133 (14)	0.0087 (16)	0.0059 (14)	0.0050 (14)
O50	0.0163 (19)	0.024 (2)	0.0172 (15)	0.0073 (16)	0.0084 (14)	0.0061 (14)
N51	0.025 (2)	0.016 (2)	0.0178 (19)	0.0041 (19)	0.0074 (18)	0.0003 (17)
C52	0.038 (3)	0.014 (3)	0.029 (3)	0.005 (3)	0.013 (2)	0.003 (2)
C53	0.026 (3)	0.025 (3)	0.026 (3)	−0.002 (3)	0.004 (2)	0.000 (2)
N54	0.014 (2)	0.021 (2)	0.0113 (17)	0.0080 (18)	0.0051 (16)	0.0044 (16)
C55	0.012 (2)	0.020 (3)	0.011 (2)	0.007 (2)	0.0008 (18)	−0.0023 (19)
C56	0.019 (3)	0.010 (3)	0.012 (2)	0.006 (2)	0.0043 (19)	0.0023 (18)
C57	0.018 (3)	0.016 (3)	0.012 (2)	0.001 (2)	0.0027 (19)	0.0011 (19)
C58	0.014 (3)	0.015 (3)	0.0090 (19)	0.007 (2)	0.0026 (18)	0.0024 (18)
C59	0.017 (3)	0.009 (3)	0.0084 (19)	0.003 (2)	−0.0013 (18)	−0.0018 (17)
C60	0.018 (3)	0.015 (3)	0.014 (2)	0.006 (2)	0.0068 (19)	0.0022 (19)
O61	0.0165 (18)	0.023 (2)	0.0173 (15)	0.0080 (16)	0.0107 (14)	0.0070 (14)
C62	0.039 (4)	0.050 (4)	0.045 (3)	0.030 (3)	0.033 (3)	0.032 (3)
C63	0.017 (3)	0.014 (3)	0.015 (2)	0.002 (2)	0.0048 (19)	0.0026 (19)
N64	0.023 (2)	0.013 (2)	0.0151 (18)	0.0059 (19)	0.0065 (17)	0.0036 (16)
C65	0.022 (3)	0.018 (3)	0.018 (2)	0.007 (2)	0.009 (2)	0.006 (2)
C66	0.029 (3)	0.020 (3)	0.020 (2)	0.005 (2)	0.010 (2)	0.004 (2)
C67	0.018 (3)	0.018 (3)	0.022 (2)	0.001 (2)	0.006 (2)	0.002 (2)
C68	0.033 (3)	0.024 (3)	0.036 (3)	0.001 (3)	0.021 (3)	0.006 (2)
C69	0.039 (3)	0.012 (3)	0.025 (2)	0.010 (3)	0.005 (2)	0.007 (2)

### Geometric parameters (Å, °)

**Table:**

O1W—H1WA	0.90 (6)	C28—H28B	0.9600
O1W—H1WB	0.90 (2)	C28—H28C	0.9600
O2W—H2WA	0.90 (6)	C29—H29A	0.9600
O2W—H2WB	0.89 (5)	C29—H29B	0.9600
O3W—H3WA	0.89 (4)	C29—H29C	0.9600
O3W—H3WB	0.90 (3)	N41—C46	1.338 (5)
O4W—H4WA	0.90 (4)	N41—C42	1.377 (6)
O4W—H4WB	0.90 (3)	N41—C47	1.473 (6)
O5W—H5WA	0.89 (4)	C42—C43	1.349 (7)
O5W—H5WB	0.89 (5)	C42—H42	0.9300
N1—C2	1.346 (5)	C43—C44	1.428 (6)
N1—C6	1.371 (6)	C43—H43	0.9300
N1—C7	1.461 (6)	C44—N54	1.342 (6)
C2—C3	1.369 (6)	C44—C45	1.427 (6)
C2—H2	0.9300	C45—C46	1.365 (6)
C3—C4	1.428 (6)	C45—S48	1.777 (4)
C3—S8	1.781 (4)	C46—H46	0.9300
C4—N14	1.339 (6)	C47—H47A	0.9600
C4—C5	1.428 (6)	C47—H47B	0.9600
C5—C6	1.351 (7)	C47—H47C	0.9600
C5—H5	0.9300	S48—O50	1.434 (3)
C6—H6	0.9300	S48—O49	1.449 (3)
C7—H7A	0.9600	S48—N51	1.617 (4)
C7—H7B	0.9600	N51—C53	1.478 (6)
C7—H7C	0.9600	N51—C52	1.482 (6)
S8—O10	1.430 (3)	C52—H52A	0.9600
S8—O9	1.440 (3)	C52—H52B	0.9600
S8—N11	1.622 (4)	C52—H52C	0.9600
N11—C12	1.463 (6)	C53—H53A	0.9600
N11—C13	1.473 (6)	C53—H53B	0.9600
C12—H12A	0.9600	C53—H53C	0.9600
C12—H12B	0.9600	N54—C55	1.426 (6)
C12—H12C	0.9600	N54—H54	0.90 (6)
C13—H13A	0.9600	C55—C60	1.375 (6)
C13—H13B	0.9600	C55—C56	1.388 (6)
C13—H13C	0.9600	C56—C57	1.387 (6)
N14—C15	1.436 (6)	C56—H56	0.9300
N14—H14	0.89 (6)	C57—C58	1.383 (6)
C15—C16	1.380 (6)	C57—H57	0.9300
C15—C20	1.378 (6)	C58—O61	1.375 (5)
C16—C17	1.385 (6)	C58—C59	1.407 (6)
C16—H16	0.9300	C59—C60	1.401 (6)
C17—C18	1.382 (6)	C59—C63	1.504 (6)
C17—H17	0.9300	C60—H60	0.9300
C18—O21	1.369 (5)	O61—C62	1.433 (5)
C18—C19	1.414 (6)	C62—H62A	0.9600
C19—C20	1.396 (6)	C62—H62B	0.9600
C19—C23	1.509 (6)	C62—H62C	0.9600
C20—H20	0.9300	C63—N64	1.526 (6)
O21—C22	1.429 (5)	C63—H63A	0.9700
C22—H22A	0.9600	C63—H63B	0.9700
C22—H22B	0.9600	N64—C69	1.493 (6)
C22—H22C	0.9600	N64—C67	1.513 (6)
C23—N24	1.528 (6)	N64—C65	1.534 (5)
C23—H23A	0.9700	C65—C66	1.511 (6)
C23—H23B	0.9700	C65—H65A	0.9700
N24—C29	1.505 (6)	C65—H65B	0.9700
N24—C27	1.522 (6)	C66—H66A	0.9600
N24—C25	1.523 (5)	C66—H66B	0.9600
C25—C26	1.510 (7)	C66—H66C	0.9600
C25—H25A	0.9700	C67—C68	1.529 (6)
C25—H25B	0.9700	C67—H67A	0.9700
C26—H26A	0.9600	C67—H67B	0.9700
C26—H26B	0.9600	C68—H68A	0.9600
C26—H26C	0.9600	C68—H68B	0.9600
C27—C28	1.514 (7)	C68—H68C	0.9600
C27—H27A	0.9700	C69—H69A	0.9600
C27—H27B	0.9700	C69—H69B	0.9600
C28—H28A	0.9600	C69—H69C	0.9600
			
H1WA—O1W—H1WB	112 (4)	H29A—C29—H29C	109.5
H2WA—O2W—H2WB	113 (5)	H29B—C29—H29C	109.5
H3WA—O3W—H3WB	111 (4)	C46—N41—C42	119.0 (4)
H4WA—O4W—H4WB	111 (4)	C46—N41—C47	121.3 (4)
H5WA—O5W—H5WB	113 (5)	C42—N41—C47	119.6 (3)
C2—N1—C6	118.4 (4)	C43—C42—N41	121.6 (4)
C2—N1—C7	121.3 (4)	C43—C42—H42	119.2
C6—N1—C7	120.3 (4)	N41—C42—H42	119.2
N1—C2—C3	122.2 (4)	C42—C43—C44	121.0 (4)
N1—C2—H2	118.9	C42—C43—H43	119.5
C3—C2—H2	118.9	C44—C43—H43	119.5
C2—C3—C4	120.7 (4)	N54—C44—C43	121.6 (4)
C2—C3—S8	115.0 (3)	N54—C44—C45	123.0 (4)
C4—C3—S8	124.2 (3)	C43—C44—C45	115.4 (4)
N14—C4—C5	122.1 (4)	C46—C45—C44	120.4 (4)
N14—C4—C3	123.0 (4)	C46—C45—S48	115.1 (3)
C5—C4—C3	114.9 (4)	C44—C45—S48	124.1 (3)
C6—C5—C4	120.7 (4)	N41—C46—C45	122.4 (4)
C6—C5—H5	119.7	N41—C46—H46	118.8
C4—C5—H5	119.7	C45—C46—H46	118.8
C5—C6—N1	122.4 (4)	N41—C47—H47A	109.5
C5—C6—H6	118.8	N41—C47—H47B	109.5
N1—C6—H6	118.8	H47A—C47—H47B	109.5
N1—C7—H7A	109.5	N41—C47—H47C	109.5
N1—C7—H7B	109.5	H47A—C47—H47C	109.5
H7A—C7—H7B	109.5	H47B—C47—H47C	109.5
N1—C7—H7C	109.5	O50—S48—O49	119.00 (19)
H7A—C7—H7C	109.5	O50—S48—N51	107.7 (2)
H7B—C7—H7C	109.5	O49—S48—N51	107.2 (2)
O10—S8—O9	119.4 (2)	O50—S48—C45	107.9 (2)
O10—S8—N11	107.1 (2)	O49—S48—C45	107.1 (2)
O9—S8—N11	107.6 (2)	N51—S48—C45	107.4 (2)
O10—S8—C3	107.0 (2)	C53—N51—C52	113.4 (4)
O9—S8—C3	107.0 (2)	C53—N51—S48	116.5 (3)
N11—S8—C3	108.3 (2)	C52—N51—S48	117.6 (3)
C12—N11—C13	113.2 (4)	N51—C52—H52A	109.5
C12—N11—S8	117.3 (4)	N51—C52—H52B	109.5
C13—N11—S8	116.3 (3)	H52A—C52—H52B	109.5
N11—C12—H12A	109.5	N51—C52—H52C	109.5
N11—C12—H12B	109.5	H52A—C52—H52C	109.5
H12A—C12—H12B	109.5	H52B—C52—H52C	109.5
N11—C12—H12C	109.5	N51—C53—H53A	109.5
H12A—C12—H12C	109.5	N51—C53—H53B	109.5
H12B—C12—H12C	109.5	H53A—C53—H53B	109.5
N11—C13—H13A	109.5	N51—C53—H53C	109.5
N11—C13—H13B	109.5	H53A—C53—H53C	109.5
H13A—C13—H13B	109.5	H53B—C53—H53C	109.5
N11—C13—H13C	109.5	C44—N54—C55	124.6 (4)
H13A—C13—H13C	109.5	C44—N54—H54	119 (3)
H13B—C13—H13C	109.5	C55—N54—H54	116 (3)
C4—N14—C15	125.7 (4)	C60—C55—C56	120.1 (4)
C4—N14—H14	110 (3)	C60—C55—N54	120.4 (4)
C15—N14—H14	124 (3)	C56—C55—N54	119.5 (4)
C16—C15—C20	120.5 (4)	C55—C56—C57	120.5 (4)
C16—C15—N14	121.1 (4)	C55—C56—H56	119.8
C20—C15—N14	118.4 (4)	C57—C56—H56	119.8
C15—C16—C17	119.8 (4)	C58—C57—C56	119.4 (4)
C15—C16—H16	120.1	C58—C57—H57	120.3
C17—C16—H16	120.1	C56—C57—H57	120.3
C16—C17—C18	120.3 (4)	O61—C58—C57	123.2 (4)
C16—C17—H17	119.8	O61—C58—C59	115.8 (4)
C18—C17—H17	119.8	C57—C58—C59	120.9 (4)
O21—C18—C17	123.9 (4)	C60—C59—C58	118.2 (4)
O21—C18—C19	115.8 (4)	C60—C59—C63	121.4 (4)
C17—C18—C19	120.3 (4)	C58—C59—C63	120.3 (4)
C20—C19—C18	118.0 (4)	C55—C60—C59	120.9 (4)
C20—C19—C23	120.0 (4)	C55—C60—H60	119.6
C18—C19—C23	121.8 (4)	C59—C60—H60	119.6
C15—C20—C19	120.9 (4)	C58—O61—C62	116.7 (4)
C15—C20—H20	119.5	O61—C62—H62A	109.5
C19—C20—H20	119.5	O61—C62—H62B	109.5
C18—O21—C22	116.3 (4)	H62A—C62—H62B	109.5
O21—C22—H22A	109.5	O61—C62—H62C	109.5
O21—C22—H22B	109.5	H62A—C62—H62C	109.5
H22A—C22—H22B	109.5	H62B—C62—H62C	109.5
O21—C22—H22C	109.5	C59—C63—N64	115.6 (4)
H22A—C22—H22C	109.5	C59—C63—H63A	108.4
H22B—C22—H22C	109.5	N64—C63—H63A	108.4
C19—C23—N24	116.0 (4)	C59—C63—H63B	108.4
C19—C23—H23A	108.3	N64—C63—H63B	108.4
N24—C23—H23A	108.3	H63A—C63—H63B	107.4
C19—C23—H23B	108.3	C69—N64—C67	110.6 (4)
N24—C23—H23B	108.3	C69—N64—C63	107.3 (4)
H23A—C23—H23B	107.4	C67—N64—C63	112.2 (3)
C29—N24—C27	110.4 (4)	C69—N64—C65	109.5 (4)
C29—N24—C25	111.2 (4)	C67—N64—C65	108.2 (3)
C27—N24—C25	108.3 (3)	C63—N64—C65	109.1 (3)
C29—N24—C23	106.6 (3)	C66—C65—N64	116.0 (4)
C27—N24—C23	109.3 (3)	C66—C65—H65A	108.3
C25—N24—C23	111.0 (3)	N64—C65—H65A	108.3
C26—C25—N24	113.8 (4)	C66—C65—H65B	108.3
C26—C25—H25A	108.8	N64—C65—H65B	108.3
N24—C25—H25A	108.8	H65A—C65—H65B	107.4
C26—C25—H25B	108.8	C65—C66—H66A	109.5
N24—C25—H25B	108.8	C65—C66—H66B	109.5
H25A—C25—H25B	107.7	H66A—C66—H66B	109.5
C25—C26—H26A	109.5	C65—C66—H66C	109.5
C25—C26—H26B	109.5	H66A—C66—H66C	109.5
H26A—C26—H26B	109.5	H66B—C66—H66C	109.5
C25—C26—H26C	109.5	N64—C67—C68	114.7 (4)
H26A—C26—H26C	109.5	N64—C67—H67A	108.6
H26B—C26—H26C	109.5	C68—C67—H67A	108.6
C28—C27—N24	115.0 (4)	N64—C67—H67B	108.6
C28—C27—H27A	108.5	C68—C67—H67B	108.6
N24—C27—H27A	108.5	H67A—C67—H67B	107.6
C28—C27—H27B	108.5	C67—C68—H68A	109.5
N24—C27—H27B	108.5	C67—C68—H68B	109.5
H27A—C27—H27B	107.5	H68A—C68—H68B	109.5
C27—C28—H28A	109.5	C67—C68—H68C	109.5
C27—C28—H28B	109.5	H68A—C68—H68C	109.5
H28A—C28—H28B	109.5	H68B—C68—H68C	109.5
C27—C28—H28C	109.5	N64—C69—H69A	109.5
H28A—C28—H28C	109.5	N64—C69—H69B	109.5
H28B—C28—H28C	109.5	H69A—C69—H69B	109.5
N24—C29—H29A	109.5	N64—C69—H69C	109.5
N24—C29—H29B	109.5	H69A—C69—H69C	109.5
H29A—C29—H29B	109.5	H69B—C69—H69C	109.5
N24—C29—H29C	109.5		
			
C6—N1—C2—C3	4.1 (7)	C46—N41—C42—C43	2.2 (7)
C7—N1—C2—C3	−173.6 (5)	C47—N41—C42—C43	−174.4 (5)
N1—C2—C3—C4	1.9 (8)	N41—C42—C43—C44	0.9 (7)
N1—C2—C3—S8	178.1 (4)	C42—C43—C44—N54	176.9 (5)
C2—C3—C4—N14	173.7 (5)	C42—C43—C44—C45	−3.6 (7)
S8—C3—C4—N14	−2.2 (7)	N54—C44—C45—C46	−177.1 (4)
C2—C3—C4—C5	−8.0 (7)	C43—C44—C45—C46	3.4 (7)
S8—C3—C4—C5	176.2 (4)	N54—C44—C45—S48	−4.7 (7)
N14—C4—C5—C6	−173.1 (5)	C43—C44—C45—S48	175.7 (3)
C3—C4—C5—C6	8.5 (7)	C42—N41—C46—C45	−2.4 (7)
C4—C5—C6—N1	−3.1 (8)	C47—N41—C46—C45	174.2 (5)
C2—N1—C6—C5	−3.5 (7)	C44—C45—C46—N41	−0.5 (7)
C7—N1—C6—C5	174.2 (5)	S48—C45—C46—N41	−173.5 (4)
C2—C3—S8—O10	41.5 (4)	C46—C45—S48—O50	−35.4 (4)
C4—C3—S8—O10	−142.5 (4)	C44—C45—S48—O50	151.9 (4)
C2—C3—S8—O9	170.5 (4)	C46—C45—S48—O49	−164.7 (4)
C4—C3—S8—O9	−13.4 (5)	C44—C45—S48—O49	22.6 (5)
C2—C3—S8—N11	−73.7 (4)	C46—C45—S48—N51	80.4 (4)
C4—C3—S8—N11	102.4 (4)	C44—C45—S48—N51	−92.3 (4)
O10—S8—N11—C12	171.9 (3)	O50—S48—N51—C53	−177.9 (3)
O9—S8—N11—C12	42.4 (4)	O49—S48—N51—C53	−48.7 (4)
C3—S8—N11—C12	−73.1 (4)	C45—S48—N51—C53	66.1 (4)
O10—S8—N11—C13	−49.7 (4)	O50—S48—N51—C52	42.5 (4)
O9—S8—N11—C13	−179.2 (3)	O49—S48—N51—C52	171.7 (3)
C3—S8—N11—C13	65.4 (4)	C45—S48—N51—C52	−73.4 (4)
C5—C4—N14—C15	7.9 (7)	C43—C44—N54—C55	−14.1 (7)
C3—C4—N14—C15	−173.8 (4)	C45—C44—N54—C55	166.3 (4)
C4—N14—C15—C16	47.5 (7)	C44—N54—C55—C60	132.6 (5)
C4—N14—C15—C20	−135.6 (5)	C44—N54—C55—C56	−50.3 (6)
C20—C15—C16—C17	3.1 (7)	C60—C55—C56—C57	−0.9 (7)
N14—C15—C16—C17	−180.0 (4)	N54—C55—C56—C57	−178.0 (4)
C15—C16—C17—C18	−0.1 (7)	C55—C56—C57—C58	0.1 (6)
C16—C17—C18—O21	179.3 (4)	C56—C57—C58—O61	179.3 (4)
C16—C17—C18—C19	−2.7 (7)	C56—C57—C58—C59	0.0 (6)
O21—C18—C19—C20	−179.5 (4)	O61—C58—C59—C60	−178.8 (4)
C17—C18—C19—C20	2.4 (7)	C57—C58—C59—C60	0.6 (6)
O21—C18—C19—C23	4.4 (6)	O61—C58—C59—C63	−2.7 (6)
C17—C18—C19—C23	−173.7 (4)	C57—C58—C59—C63	176.7 (4)
C16—C15—C20—C19	−3.4 (7)	C56—C55—C60—C59	1.5 (7)
N14—C15—C20—C19	179.6 (4)	N54—C55—C60—C59	178.6 (4)
C18—C19—C20—C15	0.7 (7)	C58—C59—C60—C55	−1.3 (6)
C23—C19—C20—C15	176.8 (4)	C63—C59—C60—C55	−177.4 (4)
C17—C18—O21—C22	−10.8 (6)	C57—C58—O61—C62	24.1 (6)
C19—C18—O21—C22	171.1 (4)	C59—C58—O61—C62	−156.6 (4)
C20—C19—C23—N24	88.3 (5)	C60—C59—C63—N64	−90.8 (5)
C18—C19—C23—N24	−95.7 (5)	C58—C59—C63—N64	93.3 (5)
C19—C23—N24—C29	−170.2 (4)	C59—C63—N64—C69	−175.4 (4)
C19—C23—N24—C27	−50.9 (5)	C59—C63—N64—C67	63.0 (5)
C19—C23—N24—C25	68.5 (5)	C59—C63—N64—C65	−56.9 (5)
C29—N24—C25—C26	−48.6 (5)	C69—N64—C65—C66	−61.7 (5)
C27—N24—C25—C26	−170.1 (4)	C67—N64—C65—C66	58.9 (5)
C23—N24—C25—C26	69.9 (5)	C63—N64—C65—C66	−178.8 (4)
C29—N24—C27—C28	−51.8 (5)	C69—N64—C67—C68	−56.0 (5)
C25—N24—C27—C28	70.2 (5)	C63—N64—C67—C68	63.7 (5)
C23—N24—C27—C28	−168.7 (4)	C65—N64—C67—C68	−175.8 (4)

### Hydrogen-bond geometry (Å, °)

**Table:**

*D*—H···*A*	*D*—H	H···*A*	*D*···*A*	*D*—H···*A*
O1W—H1WA···I4	0.91 (3)	2.60 (4)	3.489 (4)	170 (3)
O1W—H1WB···I2	0.90 (2)	2.66 (2)	3.561 (4)	176 (4)
O2W—H2WA···O5w^i^	0.90 (4)	1.98 (5)	2.799 (7)	147 (5)
O2W—H2WB···I3	0.90 (4)	2.69 (4)	3.580 (5)	175 (4)
O3W—H3WA···O1W^i^	0.90 (4)	1.91 (4)	2.781 (6)	161 (18)
O3W—H3WB···I1	0.90 (4)	2.64 (4)	3.544 (4)	178 (5)
O4W—H4WA···I4	0.90 (3)	2.75 (3)	3.613 (5)	160 (4)
O4W—H4WB···O3W^ii^	0.89 (5)	1.92 (4)	2.795 (7)	162 (2)
O5W—H5WA···I2	0.90 (4)	2.75 (5)	3.614 (5)	165 (4)
O5W—H5WB···O4w^iii^	0.90 (4)	1.94 (4)	2.809 (7)	161 (3)
N14—H14···O9	0.90 (3)	1.93 (4)	2.733 (5)	149 (4)
N54—H54···O49	0.90 (5)	2.08 (4)	2.767 (5)	133 (4)

Symmetry codes:  (i) *x*, *y*+1, *z*; (ii) *x*, *y*−1, *z*; (iii) *x*+1, *y*, *z*.
